# Purinergic signaling and cholangiocyte regeneration: a new frontier in ischemic liver injury

**DOI:** 10.3389/frtra.2025.1731982

**Published:** 2026-01-15

**Authors:** Chinedu Nwaduru, Onyekachi Emmanuel Anyagwa, Michael Fenlon, Michelle Buff, Motaz Selim, Jean Botha, Zachary Kastenberg, Michael Zimmerman

**Affiliations:** 1 Division of Transplant and Advanced Hepatobiliary Surgery, The University of Utah, Salt Lake, UT, United States; 2 Department of Surgery, New Vision University, Tbilisi, Georgia; 3 Division of Pediatric Surgery, Intermountain Medical Center, Murray, UT, United States

**Keywords:** adenosine receptors, cholangiocyte injury, ectonucleotidases, ischemia-reperfusion injury, liver transplantation, purinergic signaling, regeneration

## Abstract

Cholangiocytes—the epithelial cells lining the biliary tree—are especially vulnerable to ischemic injury, particularly in the setting of orthotopic liver transplantation (OLT). This susceptibility stems from their reliance on an arterial blood supply and limited anaerobic capacity, predisposing them to hypoxia-induced damage. While significant research has focused on hepatocellular ischemia-reperfusion injury (IRI), the specific biology of cholangiocyte injury and regeneration remains underexplored. Recent evidence highlights purinergic signaling as a key regulator*y* axis in the liver's response to ischemia. Upon hypoxic stress, extracellular ATP is released as a damage-associated molecular pattern (DAMP), activating pro-inflammatory P_2_ receptors. Enzymatic degradation of ATP by CD39 and CD73 shifts the signaling balance toward adenosine, a potent anti-inflammatory and cytoprotective molecule acting through P_1_ receptors (A_1_, A_2_A, A_2_B, A_3_). This review synthesizes emerging data on purinergic signaling in cholangiocyte biology, emphasizing its role in modulating inflammatory injury, cellular crosstalk, and regeneration. We discuss how A_2_A and A_2_B receptor pathways suppress immune-mediated damage and promote cholangiocyte proliferation, with downstream effects on IL-6 secretion, vascular remodeling, and bile duct survival. As biliary complications remain a major cause of graft dysfunction post-transplant, harnessing purinergic mechanisms may offer a novel therapeutic frontier in improving cholangiocyte resilience and overall transplant outcomes.

## 1 Introduction

The biliary epithelium is uniquely vulnerable to ischemic injuries especially in the setting of orthotopic liver transplantation (OLT), contributing as a major cause of morbidity and mortality (1). Cholangiocytes are a heterogenous group of epithelial cells that line the biliary tree (2). Their main function is the modification of bile as it is transported through the biliary tracts. These cells—cholangiocytes—rely on an intricate arterial supply, with a limited capacity for anaerobic metabolism, making them highly susceptible to hypoxia (3). With the use of marginal liver donors in clinical setting, ischemic cholangiopathy remains a serious complication, contributing to graft loss and primary non-function (1, 4). Despite progress in understanding the mechanism in hepatocellular ischemic injury and regeneration; the biology of cholangiocyte response to hypoxic stress remains unexplored.

In recent studies, purinergic signaling has emerged as a central player in the modulation of cellular stress in the liver. Following ischemia-reperfusion injury (IRI), extracellular adenosine triphosphate (ATP) is released as a damage-associated molecular pattern (DAMP), alongside other molecules of cellular injury (5, 6). This triggers the inflammatory response via P_2_ receptors (7). Subsequently, enzymatic degradation of ATP by CD39 and CD73 results in adenosine, a powerful anti-inflammatory molecule (8). This cytoprotective effect of adenosine is effected via G-coupled receptors present on cell surfaces (A_1_, A_2A_, A_2B_, A_3_) (8–10).

Though grossly underexplored, emerging studies suggest a critical role of purinergic signaling—particularly the CD39/73 adenosinergic pathway- in cholangiocyte response to stress, proliferation and survival following ischemic insults. This review synthesizes the current understanding of purinergic signaling in the context of ischemic liver injury, focusing on its possible implications in cholangiocyte biology and the potential for clinical intervention.

## 2 Cholangiocyte injury and repair during ischemia

Cholangiocytes, the epithelial cells lining the intra- and extrahepatic bile ducts, are essential for bile formation, modification and secretion. They receive their blood supply via the peribiliary plexus, a dense 3D network of uniformly sized vessels surrounding the bile ducts (11). This close anatomical relationship facilitates dynamic crosstalk that support normal cholangiocyte function and may contribute to dysfunction in disease states (12). In cholangiopathies, the primary cellular target is the biliary epithelium—specifically cholangiocytes. Among these, intrahepatic cholangiocytes lining the small branches of the biliary tree are the earliest to respond to injury (13, 14). Most disease states of the biliary tree involve similar pathological mechanisms—such as increased cholangiocyte proliferation, apoptosis, as well as pro-fibrotic and inflammatory secretions (15, 16).

### 2.1 Pathophysiology of ductular reaction and fibrosis

**Figure 1:**
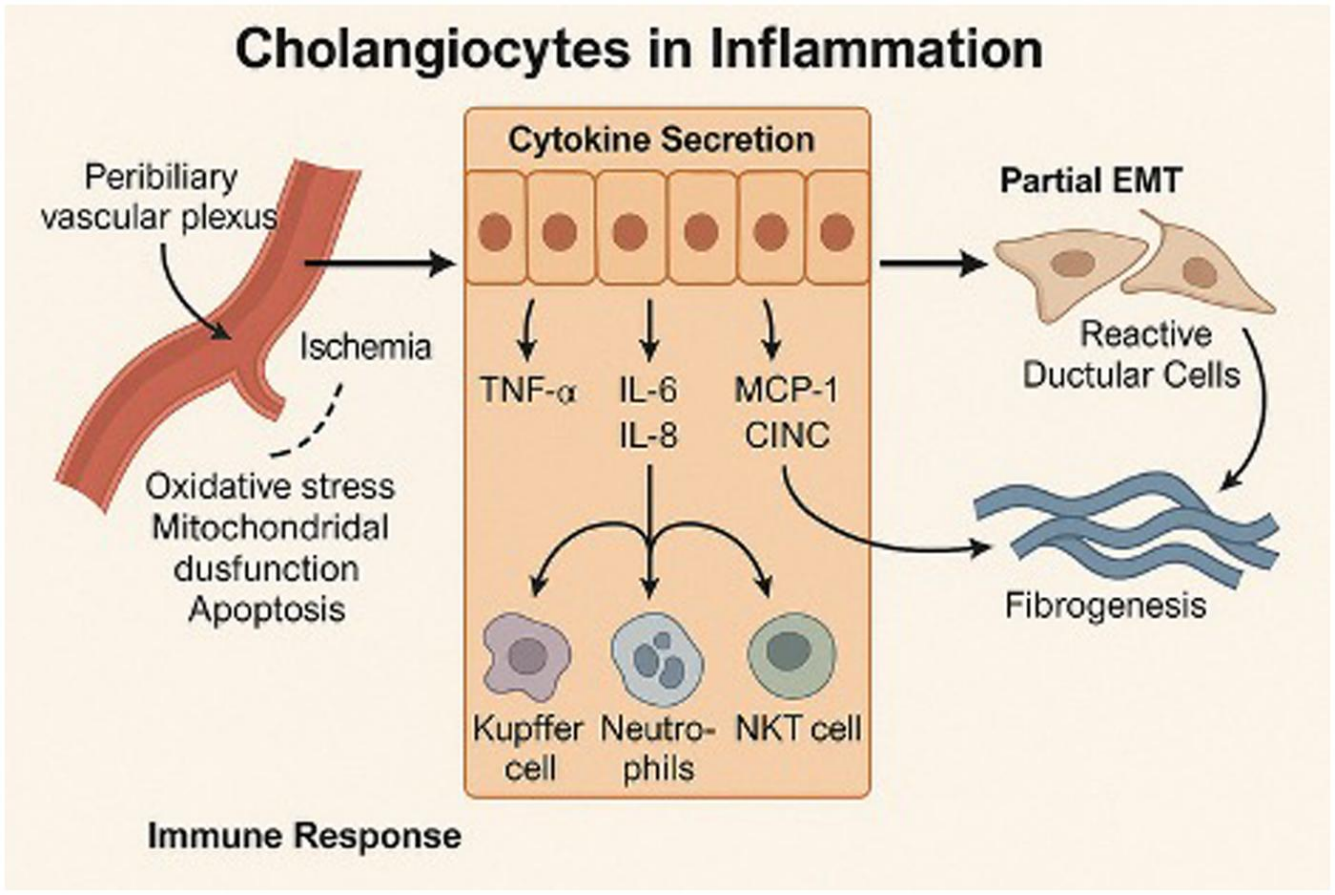
Cholangiocytes in inflammation. Ischemia of the peribiliary vascular plexus triggers oxidative stress, mitochondrial dysfunction, and apoptosis, leading to activation of cholangiocytes. These cells secrete pro-inflammatory cytokines and chemokines—such as il-6, IL-8—which recruit immune cells including Kupffer cells, neutrophils, and NKT cells. Concurrently, cholangiocytes undergo partial epithelial—to-mesenchymal transition (EMT), forming reactive ductular cells that contribute to periductal fibrogenesis.

Central to biliary repair is inflammation: persistent injury triggers an immune response that promotes tissue repair, characterized by periductal fibrosis and progression to biliary cirrhosis (17). This response, called ductular reaction, comprises of activated cholangiocytes, immune cells and mesenchymal cells. Upon exposure to various insults, including ischemic stimuli, cholangiocyte exhibit remarkable plasticity, transitioning into a reactive ductular cell (RDC) phenotype (18). These RDCS adopt a pro-inflammatory and pro-fibrotic profile, secreting several cytokines such as tumor necrosis factor-α (TNF-α), interleukins -6 and 8 (IL-6, IL-8), and chemokines like monocyte chemotactic protein-1 (MCP-1) (19, 20). These mediators facilitate the recruitment of immune and mesenchymal cells that contribute to biliary remodeling (Figure 1). This inflammatory microenvironment is further shaped by complex interactions between RDCs and various resident and recruited cells, including hepatic stellate cells (HSCs), portal fibroblasts, innate and adaptive immune cells (14, 21, 22). Concurrently, angiogenesis is actively promoted through the secretion of factors such as vascular endothelial growth factor (VEGF), endothelin-1 and transforming growth factor-β2 (TGF-β2) (20, 23). While phenotypically like liver progenitor cells, RDCs primarily mediate repair rather than regeneration and are closely linked to fibrogenesis (24). This is partly because RDCs lose their identity as epithelial cells, with partial acquisition of mesenchymal traits, contributing to fibrosis (25). Collectively, these responses culminate in progressive portal fibrosis.

### 2.2 Role of iNOS and inflammatory mediators in biliary injury

Among the downstream effects of inflammatory stress, inducible nitric oxide synthase (iNOS) plays a critical role in mediating cholangiocyte dysfunction. Pro-inflammatory cytokines and lipopolysaccharides (LPS) from bacterial cell wall induce iNOS expression in cholangiocytes, leading to nitric oxide (NO) and reactive nitrogen species (RNOS) production (26). These species disrupt the cAMP-dependent secretion by nitrosylating key transport proteins and impair bile hydration leading to cholestatic injury (26, 27). iNOS and nitrotyrosine expression observed in LPS-treated murine models and patients with primary sclerosing cholangitis (PSC) confirm this mechanism *in vivo* (27). As iNOS is regulated by NF-κB, this pathway links inflammation to cholangiocyte dysfunction and identifies iNOS inhibition as a potential therapeutic target. Beyond intrinsic injury responses, activated cholangiocytes engage in dynamic communication with immune cells, shaping the inflammatory landscape of biliary disease.

### 2.3 Cholangiocyte activation and immune crosstalk

Crosstalk between activated cholangiocytes and immune cells, particularly T cells and macrophages, plays a central role in shaping the inflammatory microenvironment. Cholangiocytes express adhesion molecules such as intercellular adhesion molecule 1 (ICAM-1) and vascular cell adhesion molecule 1 (VCAM-1); and other immune checkpoint ligands [for instance, programmed cell death 1 ligand 1 (PD-L1)] that facilitate direct interactions with t cells (28–30). Chemokines such as CXCL16 and CCL20 recruit and position effector T cells at sites of biliary injury (16, 28). This interaction is especially relevant in autoimmune liver diseases like PSC and primary biliary cirrhosis (PBC), where persistent T cell-mediated cholangiocyte damage is implicated in pathogenesis (31). Simultaneously, cholangiocytes attract monocytes through chemokines like CCL2 and IL-1, influencing macrophage activation, cytokine release, and fibrogenic signaling cascades involving hepatic stellate cells and portal myofibroblasts (15, 16, 32)..

The inflammatory biliary microenvironment is further characterized by elevated levels of IL-8, largely secreted by cholangiocytes, which serves as a chemoattractant for both monocytes and neutrophils (33). Proteins associated with neutrophil activation [for example, S100A8, S100A9, S100A12 and monocyte morphogenetic protein-9 (MMP9)] are notably enriched in the bile of PSC patients (34–36). Moreover, cholangiocyte-derived cytokines like IL-6 not only sustain immune activation but also promote cholangiocyte proliferation through autocrine signaling (37). Over time, this persistent inflammatory crosstalk may contribute to carcinogenesis, facilitating cholangiocarcinoma development in chronically inflamed biliary tissues (38). This sets the stage to explore how purinergic signaling within the biliary tree further regulates these inflammatory and reparative responses.

### 2.4 Cholangiocyte regeneration: beyond hepatocytes

The liver's regenerative capacity has traditionally been attributed to the self-renewal of hepatocytes; however, recent evidence highlights an important role of cholangiocyte proliferation and trans-differentiation in enhancing liver repair. Growth factors such as hepatocyte growth factor (HGF) and ligands of the epidermal growth factor receptor (EGFR)—including amphiregulin (AREG)- serve as potent mitogens promoting hepatocellular growth (39, 40). Studies have shown that cholangiocytes can secrete HGF and other growth factors under pathological conditions and in cholangiocytes with tumor suppressor gene deficiencies (41, 42). Additionally, proinflammatory cytokines like TNF-α and IL-6 are critical regulator of regenerative signaling, with their deficiency impairing hepatocyte proliferation pathways (43)..

Beyond supporting hepatocyte regeneration, cholangiocytes initiate autocrine and paracrine programs to sustain their own proliferation during injury as previously mentioned. Factors like IL-8, found elevated in the bile of PSC patients play crucial role in this scenario (39, 44). Bile acids like taurocholate further enhance cholangiocyte survival and regeneration by upregulation VEGF-A and VEGF-C (45). Furthermore, cholangiocytes secrete nerve growth factor (NGF), which stimulates their own growth through ERK1/2-signaling pathway, with *in vivo* studies confirming that NGF neutralization impairs biliary epithelial cell proliferation after bile duct ligation (46).

Recent findings further illustrate a complex network of cellular crosstalk in cholangiocyte-driven regeneration. These studies collectively propose that bile duct repair is regulated through a dynamic balance of stimulatory and inhibitory factors, involving both cholangiocyte-derived signals and contributions from neighboring immune and stromal cells (15, 47). This evolving understanding underscores the multifactorial nature of cholangiocyte regeneration in the context of liver injury.

Indeed, despite the critical role of cholangiocyte in maintaining biliary integrity, relatively few studies have directly investigated the mechanisms underpinning biliary epithelial regeneration. Much of our current understanding derives indirectly from broader research into liver regeneration, where the contribution of cholangiocytes has been inferred through their participation in hepatic progenitor cell activation, ductular reactions and repair of periportal injury. Given the importance of epithelial survival and regeneration, understanding the regulatory networks—particularly purinergic pathways—that modulate cholangiocyte fate becomes critical.

### 2.5 Linking cholangiocyte vulnerability to ischemic and immune cell-mediated injury

To appreciate the dual effects of the purinergic signaling on cholangiocyte physiology, it is crucial to understand that cholangiocytes possess several structural and functional traits that uniquely sensitize them to immune-mediated injury. First, their arterial supply predisposes them to ischemic stress, which amplifies the release of ATP and inflammatory cytokines. Second, cholangiocytes express high levels of adhesion molecules (ICAM-1, VCAM-1), chemokines (CXCL16, CCL20), and immune checkpoint ligands (PD-L1, enabling direct interactions with infiltrating T cells and macrophages. These interactions promote targeted cytotoxicity and sustained cytokine exposure, particularly from natural killer T (NKT) cells and neutrophils, which accumulate around bile ducts during ischemic and autoimmune injury. In addition, cholangiocytes exhibit a heightened propensity for loss of polarity, secretory dysfunction, and partial epithelial-mesenchymal transition (BMT) when subjected to ATP-driven inflammatory stress (48). This susceptibility amplifies the damaging effects of purinergic signaling on epithelial integrity, linking innate immune activation directly to cholangiocyte-specific pathological responses.

While much of the existing literature on cholangiocyte injury derives from chronic immune-mediated cholangiopathies such as PSC and PBC, ischemia-driven biliary injury represents a much distinct entity. In ischemic cholangiopathy, which is commonly observed following donation-after-circulatory-death (DCD) transplantation, prolonged cold ischemia, or marginal graft use (49–52); cholangiocyte injury is primarily initiated by hypoxia, microvascular collapse of the peribiliary plexus, and ATP-driven sterile inflammation rather than sustained autoimmunity (3). Clinically, this manifests as non-anastomotic strictures, diffuse biliary necrosis, and impaired ductal regeneration (3).

In contrast, chronic cholangiopathies are characterized by persistent immune cell infiltration, cytokine-driven epithelial activation, and progressive ductular reaction over months to years as previously described. Although overlapping inflammatory mediators are involved, IRI is temporally acute, metabolically driven, and highly dependent on purinergic signaling during reperfusion. Distinguishing these mechanisms (ischemic vs. immune-mediated injuries) is essential, as purinergic pathways, especially the balance between ATP-mediated P_2_ signaling and adenosine-mediated P_1_ signaling are uniquely positioned to influence early cholangiocyte survival and repair following ischemic insult rather than chronic immune remodeling.

## 3 Overview of purinergic signaling in the liver and biliary tree

### 3.1 Overview of P1 and P2 receptors

Adenosine and ATP regulate diverse hepatic functions through purinergic signaling, which involves two main receptor families: P_1_ receptors (which bind adenosine) and P_2_ receptors (which bind nucleotides like ATP and uridine diphosphate) (53). Four subtypes of adenosine receptors—A_1_, A_2_A, A_2_B, A_3_- have been identified in the liver, including cholangiocytes (54). In cholangiocytes, A2B receptor activation has been shown to promote IL-6 secretion and support cell survival during injury (55). The specific roles of adenosine receptors in modulating hepatic blood flow and epithelial function remain under investigation, but their presence however, on vascular endothelium and biliary epithelium suggests they may play a significant role in hypoxia adaptation and inflammation (56).

### 3.2 ATP as a DAMP and the CD39/CD73 adenosinergic axis

Ischemic stress from hypoxic conditions to the liver triggers the release of purine nucleotides, including ATP and nucleosides into the hepatic microenvironment. Extracellular ATP, acting as DAMP causes direct cellular injury and inflammation—causing the release of intracellular ATP from dying cells, which further increases the inflammatory cascade—a vicious cycle (16). This ATP-driven damage pathway is counter-regulated by ectonucleotidases—notably CD39 (nucleoside triphosphate diphosphorylase—1) and CD73 (5′-ectonucleotidase)—which sequentially break down ATP/ADP to adenosine monophosphate (AMP) and then finally to adenosine (8). The resulting shift from ATP to adenosine has important protective effects: while ATP (acting on P_2_ receptors) tend to promote inflammation and cell death; adenosine (acting on P_1_ receptors) is largely immunomodulatory and cytoprotective (57).

### 3.3 Purinergic regulation of cholangiocytes

Under physiologic conditions, cholangiocytes continuously release ATP into bile and interstitial spaces, contributing to the local extracellular nucleotide pool (53). ATP release occurs constitutively and is enhanced by stimuli such as cellular swelling and mechanical stress (58). In cultured cholangiocytes, ATP release is polarized, with higher concentrations at the apical surface (approximately fivefold greater than the concentrations in the basolateral chamber) matching the distribution of apical P_2_ receptors that regulate biliary chloride secretion (58, 59). These findings support a model in which ATP released from hepatocytes and cholangiocytes act in an autocrine and paracrine fashion to regulate biliary function.

**Figure 2:**
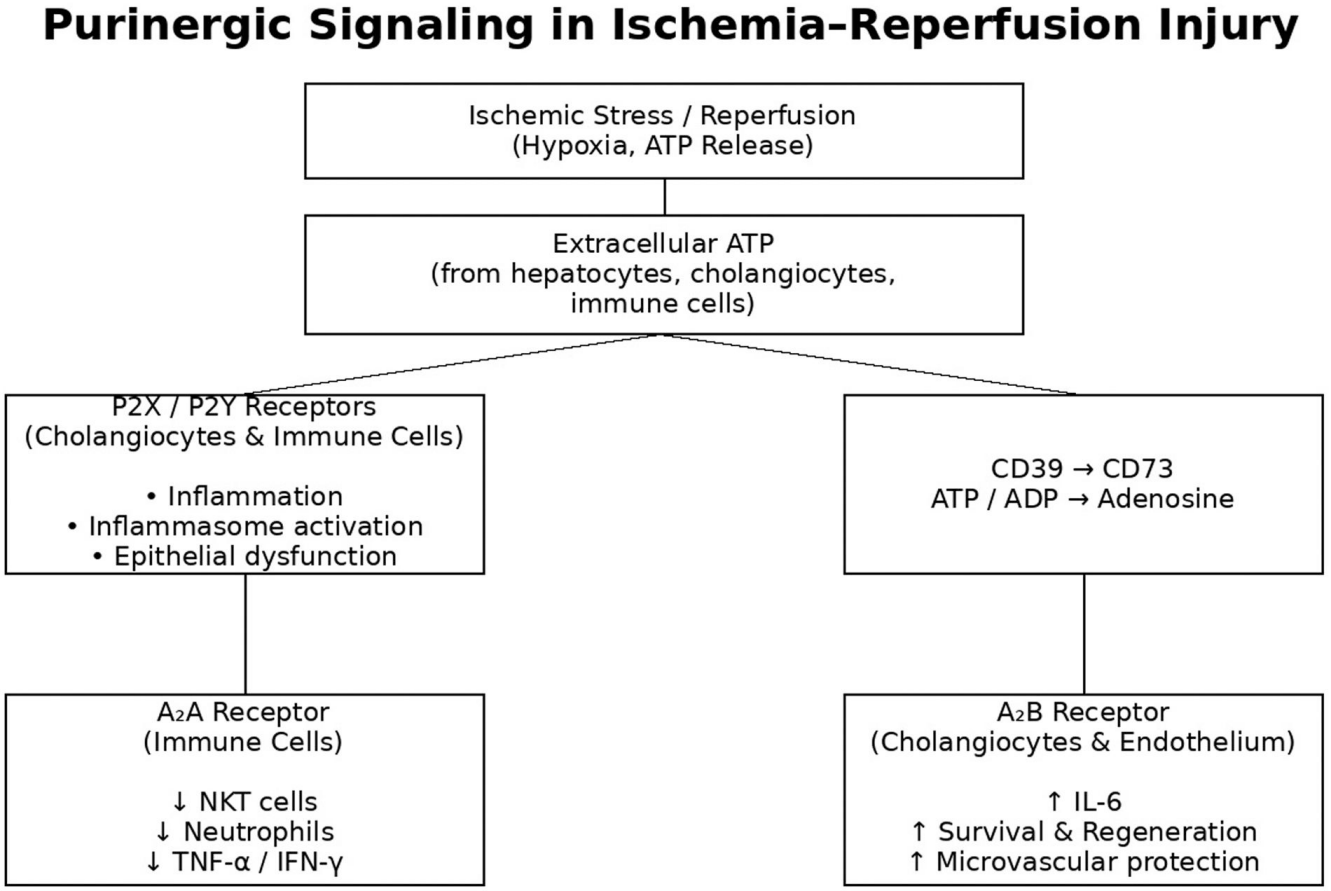
Purinergic signaling during ischemia–reperfusion injury in the biliary microenvironment. Ischemic stress induces extracellular ATP release from injured hepatocytes, cholangiocytes, and immune cells. ATP activates P2X and P2Y receptors on cholangiocytes and immune cells, promoting inflammation, inflammasome activation, and epithelial dysfunction. Sequential hydrolysis of ATP and ADP by CD39 and CD73 generates extracellular adenosine, shifting signaling toward P1 receptor activation.

Purinergic signaling plays a pivotal role in hepatocyte and cholangiocyte regeneration by promoting proliferation and cytoprotection against injury (Figure 2) (57). Following partial hepatectomy (PH), one of the most studied models of liver regeneration, mechanical stress triggers rapid ATP release into the extracellular space, initiating cell cycle entry (60). P_2_X_4_ receptors support regeneration by regulating biliary homeostasis, while P_2_X_4_ deficiency results in increased necrosis, cholestasis and delayed recovery (61). Additionally, P_2_Y_2_ receptors contribute by modulating sinusoidal endothelial cells to enhance HGF and IL-6 secretion through VEGF receptor 2 phosphorylation, further supporting regenerative processes (57, 62). Loss of CD39 impairs endothelial integrity, reduces hepatocyte regeneration, and worsens survival outcomes (63). Natural killer (NK) cells participate by hydrolyzing ATP and enhancing cytotoxicity, with apyrase administration shown to augment hepatocyte proliferation through P_2_X_3_- and P_2_Y_1_-mediated pathways (64). Furthermore, PH mobilizes CD39+ hematopoietic stem cells from the bone marrow, which contribute to regeneration through CD39- mediated ATP breakdown and activation of A_2_A receptor signaling (65).

In addition to adenosine receptors—P_2_X and P_2_Y receptors, both expressed on cholangiocytes, mediate responses to extracellular ATP (59, 66). For example, these cells express P_2_X_4_ and P_2_X_5_ receptors that, when activated by ATP, open apical chloride (Cl^−^) channels to drive bicarbonate-rich bile secretion (66). P_2_X_7_ receptors may further regulate cell fate and inflammatory signaling (54). On the other hand, P_2_Y receptors have distinct effects on cholangiocyte biology: P_2_Y_2_ triggers Na^+^/H^+^ exchange to promote bicarbonate secretion (67). Interestingly, P_2_Y_2_, P_2_Y_4_, and P_2_Y_6_ receptor activation have been shown to downregulate cholangiocyte IL-6 expression (68). In contrast, P_2_Y_11_ activation in cholangiocytes induces Ca^2+^ and cAMP signaling that increases IL-6 secretion- driving cholangiocyte proliferation and survival during injury (68). This adenosine-IL-6 axis has been shown to influence how these cells cope with hypoxic injury, as discussed subsequently.

Notably, despite extensive work examining purinergic signaling in various tissue and cell types including hepatocytes and endothelial cells, there remains a striking scarcity of studies that directly interrogate its impact on cholangiocytes. This gap underscores the timeliness of the present review, which highlights emerging evidence and anticipates a growing need for cholangiocyte-focused investigations into purinergic regulation during ischemic injury.

### 3.4 CD39 and CD73: regulating hepatobiliary ischemia

The CD39-CD73 adenosinergic pathway is a critical molecular mechanism in controlling purinergic signaling hepatobiliary ischemia. Ischemic preconditioning (IP) experiments have demonstrated hepatic upregulation of these molecules as a cytoprotective strategy. Brief cycle of ischemia or systemic hypoxia induce transcription of ENTPD1 (CD39) via Sp1 and hypoxia-inducible factors (HIFs), leading to the upregulation of CD73 (69, 70). This results in accelerated breakdown of ATP to adenosine during subsequent prolonged ischemia (70). *in vivo* studies in *CD39^−^/CD73^−^* mice show loss of cytoprotection conferred by IP—thus leading to increased susceptibility to ischemia-reperfusion injury (71). Conversely, Hart et al. found that hepatic IP in wild-type mice greatly increased CD39 expression and adenosine levels, which correlated with reduced liver injury on reperfusion (69). These findings establish the importance of extracellular ATP hydrolysis as a required step in liver and biliary ischemic tolerance, by preventing ATP/P_2_X mediated cell death by generating protective adenosine.

Consistent with this, transgenic mice overexpressing CD39 are remarkably resistant to I/R injury (72). Protection in CD39^−^ overexpressing grafts was linked to an altered immune response—with fewer resident CD4^+^ Tcells in the liver after reperfusion (72). This suggests that beyond direct biochemical effects, the adenosine-rich environment modulated by CD39 can dampen T-cell mediated damage in transplantation. While enzymatic regulation of extracellular nucleotides is pivotal, the downstream effects are ultimately mediated through specific adenosine receptors, each orchestrating distinct cellular response.

### 3.5 Adenosine receptors in cholangiocyte ischemic injury

The adenosine A_2_A receptor is a G_s_-coupled receptor widely expressed on immune cells, endothelium, and other liver cells (57). A_2_A signaling is strongly anti-inflammatory and has been shown to modulate I/R injury in many contexts (73). In warm liver I/R models, administration of a selective A_2_A agonist during reperfusion dramatically reduces ischemic injury. For example, the administration of an A_2_A agonist at the onset of reperfusion in mice models showed significantly lower transaminase levels, reduced neutrophil sequestration in the liver, and less TNF-α/IFN-γ expression (74). A key target appears to be resident hepatocyte T cells with natural killer features (NKT cells), which are early drivers of ischemic injury (75). During warm ischemia, NKT cells become activated and produce IFN-γ, exacerbating hepatocyte and cholangiocyte injury (75). This underscores that A_2_A signaling on immune cells potently suppresses the inflammatory cascade of reperfusion.

Importantly, cholangiocyte injury in I/R is immune mediated, highlighting the importance of A_2_A's immunosuppressive effect on bile duct injury. There is some evidence that these receptors on hepatic stellate cells and other non-parenchymal cells promote anti-apoptotic and pro-proliferative pathways that could benefit cholangiocytes' repair (76). In stellate cells, A_2_A activation raises cAMP/PKA signaling, reducing cellular contractility and death (77). This may translate into better sinusoidal blood flow and more supportive milieu for biliary epithelial survival. However, much is yet to be explored regarding this direct cytoprotective effect on biliary cells.

#### 3.5.1 Role of A2B receptors: promoting cholangiocyte survival

The adenosine A_2_B receptor is a low-affinity receptor that becomes activated in conditions of high extracellular adenosine, especially during ischemia or hypoxia when CD39/CD73 activity is upregulated (56). These receptors are expressed on numerous cells in the liver, including sinusoidal endothelium, and likely cholangiocytes and stromal cells (57). While A_2_B was once less studied than A_2_B, evidence now points to a critical role in ischemic live injury and specifically in cholangiocyte response to injury.

A landmark study by Chouker et al. demonstrated that the protection afforded by *in vivo* hypoxic preconditioning depended entirely on A_2B_ receptors (78). Brief hypoxia in mice led to a doubling of liver adenosine levels and significant attenuation of I/R injury: lower transaminases, TNF-α, and IL-6 levels—compared to non-preconditioned controls (78). However, when the same protocol was applied to A_2_B knockout mice—or wild type mice given an A_2_B antagonist—the protective effect of hepatic preconditioning was lost (78). This was the first direct evidence that A_2_B signaling is a mediator of liver ischemic tolerance. Mechanistically, A_2_B receptors on liver endothelial cells can enhance post-ischemic microvascular injury by promoting nitric oxide production and barrier function (56). A_2_B signaling also influences immune cells; for example, A_2_B activation on Kupfer cells tend to shift them toward an anti-inflammatory phenotype, reducing the production of TNF-α (79). They also reduce activation of adaptive immune cells on hepatic antigen presenting cells (APCs) such as dendritic cells (80). Table 1 contrasts A_2_A-mediated immunomodulation with A_2_B-driven cholangiocyte trophic signaling and microvascular protection. Table 2 summarizes the dominant purinergic receptors and ectonucleotidases relevant to cholangiocyte injury and regeneration, highlighting their principal cellular targets and functional outcomes.

**Table 1:** Functional comparison of A2A and A2B adenosine receptors in ischemic biliary injury.

Characteristic	A_2_A Receptor	A_2_B Receptor
Affinity for adenosine	High	Low (activated during ischemia)
Primary cell targets	NKT cells, neutrophils, T cells	Cholangiocytes, endothelium, Kupffer cells
Dominant function	Immunosuppression	Cytoprotection and regeneration
Key downstream effect	⇓ IFN-γ, ⇓ TNF-α, ⇓ neutrophil infiltration	⇓ IL-6, ⇓ epithelial survival, ⇓ microvascular integrity
Role in cholangiocytes	Indirect (immune-mediated protection)	Direct (autocrine IL-6 signaling)
Therapeutic implication	Limit reperfusion inflammation	Promote biliary survival and repair

NKT cell, natural killer T cell; IFN-γ, Inteferon-gamma; TNF-α, tumor-necrosis factor—alpha; IL-6, Interleukin-6.

**Table 2:** Key purinergic receptor and ectonucleotidases in cholangiocyte injury and regeneration.

Receptor/ Enzyme	Major Cellular Targets	Dominant Function	Role in cholangiocyte injury/repair
P_2_X_4_/P_2_X_5_	Cholangiocytes	Ion channel activation	Regulate biliary secretion and epithelial homeostasis.
P_2_X_7_	Kupffer cells, immune cells	Inflammasome activation	Promotes inflammation and fibrogenesis during sustained injury.
P_2_Y_2_/P_2_Y_4_/P_2_Y_6_	Cholangiocytes, endothelium	G-protein signaling	Modulate secretion and suppress IL-6.
P_2_Y_11_	Cholangiocytes	cAMP/Ca^2+^ signaling	Induces IL-6 and supports epithelial survival.
A_2_A receptor	NKT cells, neutrophils, T cells	Immunosuppression	Limit immune-mediated cholangiocyte injury during reperfusion.
A_2_B receptor	Cholangiocytes, endothelium	Cytoprotection, regeneration	Drives IL-6 trophic axis and microvascular recovery.
CD39 (ENTPD1)	Endothelium, immune cell	ATP/ADP hydrolysis	Reduces ATP-driven inflammation, promotes adenosine generation.
CD73	Endothelium, epithelium	AMP to adenosine conversion	Amplifies adenosine-generated protection.
NTPDase2	Portal fibroblasts	Local ATP regulation	Supports ductular reaction and cholangiocyte proliferation.

NKT cell, natural killer T cell; cAMP, cyclic adenosine monophosphate; ATP, adenosine triphosphate; ADP, adenosine diphosphate; AMP, adenosine monophosphate; IL-6, Interleukin-6.

### 3.6 Dual functions of IL-6 in cholangiocyte biology

IL-6 is a multifunctional cytokine that regulates growth, differentiation, and survival in a cell-type-specific manner, and cholangiocytes both produce and respond to IL-6. Under basal conditions, cholangiocytes secrete IL-6, but production increases markedly in response to inflammatory stimuli such as LPS, TNF-α, and IL-1β (81). In cholangiocyte physiology, IL-6 occupies a paradoxical role: it is both pro-regenerative and potentially oncogenic. During acute ischemic injury, IL-6 acts as a trophic cytokine that promotes cholangiocyte proliferation, enhances survival pathways, and supports early epithelial repair through STAT3 activation (82). This regenerative response is especially pronounced when IL-6 is induced downstream of purinergic A_2_B receptor signaling, which amplifies cytoprotective programs during hypoxia. However, in chronic inflammatory states including primary biliary cirrhosis, bile duct obstruction, and viral hepatitis, cholangiocyte IL-6 expression is dysregulated, and rodent models demonstrate that IL-6 is essential for cholangiocyte proliferation after bile duct ligation or partial hepatectomy (83)..

Additionally, A_2_B receptors play a unique role in autocrine IL-6 signaling. Dranoff and colleagues described an “adenosine-IL-6 axis” in cholangiocytes where adenosine, generated extracellularly by CD39/CD73 stimulates cholangiocyte A_2_B receptors (55). This activation leads to a Ca^2+^-dependent induction of IL-6 mRNA and protein secretion (55). IL-6 is a trophic factor for cholangiocytes known to promote their survival and proliferation during injury and cirrhosis (84). *In vitro*, human cholangiocyte cell lines exposed to A_2_B agonist showed robust IL-6 release whilst mice cells deficient in A_2_B had impaired IL-6 upregulation in bile ducts (55). Functionally, A_2_B deficient mice experienced worse outcomes in a biliary injury model, showing significantly higher mortality than wild type controls despite similar levels of fibrosis and inflammation (55). This suggests the loss of cholangiocyte IL-6 signaling left the bile ducts susceptible to cellular damage. Thus, A_2_B receptors on cholangiocytes act as sensors of extracellular adenosine that kick-start a protective IL-6 response. In I/R settings, this could translate to cholangiocytes helping themselves and neighboring cells survive by secreting IL-6 when adenosine levels arise. Malignant cholangiocytes also overproduce IL-6 and respond to it with enhanced proliferation, suggesting an autocrine or paracrine mechanism that contributes to tumor growth (83). Notably, IL-6 expression tends to decrease as cholangiocarcinoma becomes less differentiated, implying that advanced tumors may become less dependent on IL-6 driven signaling (37). Overall, IL-6 plays a central role in both physiologic biliary repair and pathological cholangiocyte expansion, including carcinogenesis.

Beyond IL-6, A_2_B receptors may have other beneficial effects: A_2_B activation is linked to improved barrier function in endothelia and reduced leucocyte adhesion and infiltration (85, 86). There is also evidence in other organs that parenchymal A_2_B signaling supports post-ischemic metabolic recovery and blood flow. For instance, in renal I/R, A_2_B receptor on renal tubules helped restore peritubular perfusion and limit injury (87). Therefore, it is plausible that in the liver, A_2_B on cholangiocytes and other cells contribute to maintaining the integrity of the bile duct epithelium and peribiliary capillaries during perfusion.

### 3.7 Experimental models highlighting purinergic mechanisms in the liver and biliary tree

Cellular models provide mechanistic insight into how purinergic signaling affects cholangiocytes under ischemic stress. When ATP is acutely depleted in cholangiocytes, it triggers major cellular disruptions. For example, a study showed ATP depletion in isolated rat cholangiocytes caused marked internalization of membrane proteins, disrupting the polarized domain of the cells (88). This mirrors the epithelial dysfunction seen in ischemic bile ducts: loss of membrane transporters and tight junctions. It underscores that maintaining ATP levels is crucial for cholangiocyte structure and function. If ischemia cannot be avoided, as in the case of orthotopic liver transplant and hepatic resection; then harnessing purinergic signaling—to minimize ATP wasteful signaling and instead engage adenosine pathways—is a logical approach. In another study, human cholangiocyte cell lines (H69 cells) were used to demonstrate that adenosine analogs acting on A_2_B receptors caused IL-6 secretion via Ca^2+^ signaling (cholangiocyte IL-6 axis) as previously discussed (55). This provides a clear mechanism by which cholangiocytes sense the adenosine surge after ischemia.

Other studies have shown that A_2_B receptor agonists (such as adenosine analogs or specific drugs like ATL-146e) given at reperfusion dose-dependently reduce liver IRI, cutting peak transaminase levels and improving histology (74, 89). These effects were lost in A_2_A-knockout animals. Mice treated with A_2_A agonists also show lower neutrophil accumulation in liver tissue and reduced expression of inflammatory genes (89). This was further elaborated in larger animal models of liver transplantation (90). Lappas et al. pinpointed NKT cells as an A_2_A-sensitive driver of injury: CD1d-deficient mice had less I/R injury, and adoptive transfer of normal NKT cells restored injury, but transfer of A_2_A-agonist-treated NKT cells did not, indicating A_2_A activation on NKT cells blunts their ability to cause damage (75). Another interesting finding is that if NKT cells are activated prior to ischemia, they can secrete IL-13 and protect the liver from subsequent IRI in an A_2_A dependent manner (91). This implies that adenosine signaling through A_2_A receptors can shift NKT cells to a hepatoprotective phenotype under certain conditions. All these immune insights are relevant to cholangiocytes, because neutrophils and NKT cells are known to target bile ducts in ischemic and inflammatory cells (16). A_2_A agonism could mitigate these attacks.

Pommey et al. used a mouse OLT model with prolonged cold storage to study biliary injury—with donor livers overexpressing CD39 showing better graft function and less biliary necrosis than wild type grafts (72). This suggests that in cold ischemia (like in actual transplant), conventional T cells contribute to bile duct injury, and high adenosine environment (via CD39/CD73 pathway) helps prevent T cell-mediated cholangiocyte injury. Another study found that NTPDase2, another ectonucleotidase—expressed by portal fibroblasts is important in biliary response to injury, promoting ductular reaction and cholangiocyte proliferation (92). This study hints that purinergic signaling in the cholangiocyte microenvironment can influence how well these cells regenerate after injury, including ischemic injury. Together, these insights underscore several promising targets within the purinergic cascade that may offer novel therapeutic strategies to enhance biliary repair and mitigate ischemic injury.

### 3.8 When good becomes bad: purinergic signaling and fibrosis in cholangiocytes

Importantly, while adenosine signaling typically confers anti-inflammatory and cytoprotective effects, certain purinergic pathways can paradoxically promote fibrosis under chronic or dysregulated conditions in cholangiocytes. For instance, persistent activation of P_2_X_7_ receptor triggers inflammasome assembly, leading to IL-1β release and downstream stellate cell activation, both of which potentiate fibrogenesis (93, 94). Similarly, ATP enrichment in the microenvironment, which is very common in sustained epithelial injury, can propagate ductular reaction and stromal activation (95).

Conversely, although CD39/CD73 activity generally shifts signaling toward anti-fibrotic adenosine, prolonged adenosine exposure can activate A_2_A receptors of hepatic stellate cells, promoting survival and proliferation of these fibrogenic cells (76). Thus, the net effect of purinergic signaling on fibrosis depends on the duration of injury and relative contributions of P_2_ vs. P_1_ receptor activation. Acute ischemia tends to favor anti-inflammatory adenosine pathways, while chronic ATP-rich injury states may skew towards fibrosis.

## 4 Therapeutic implications and future directions

Accumulating evidence from basic and translational research hints that modulating purinergic signaling can protect cholangiocytes during ischemic liver injury. Given their robust anti-inflammatory effects, A_2_A agonists are promising candidates to reduce reperfusion injury, as pre-clinical animal models have demonstrated a promising efficacy. Drugs like regadenoson, a FDA-approved A_2_A agonist for cardiac imaging could be repurposed at the time of liver reperfusion (96). Lau et al. demonstrated the safe administration of regadenoson in a pilot study for lung transplantation, paving the way for trials in OLT or major liver surgeries (97). Enhancing A_2_B signaling might prove a bit trickier, as there are no selective A_2_B agonists widely available clinically. However, leveraging the body's natural physiology, for instance, hypoxic preconditioning or hypoxia inducible factor- 1 alpha (HIF-1α) stabilization-might be a way to engage this receptor. Some studies suggest that ischemic preconditioning in mice and humans can improve outcomes (78, 98). this could occur partially via increasing adenosine A_2_B receptor. Any A_2_B-based therapy would aim to bolster the cholangiocyte IL-6 survival axis and improve microvascular recovery.

Another compelling approach instead of administering adenosine (which is rapidly metabolized)—is to administer enzymes that generate adenosine from released ATP on-site. Soluble CD39 (apyrase) could be infused into the liver circulation to degrade ATP during cold storage and early reperfusion. There is strong experimental evidence from mice treated with apyrase, which showed dramatic reduction in liver IRI (69). a human recombinant is commercially unavailable, but efforts in other fields including oncology are exploring CD39-based biologics.

P_2_ receptor antagonists can prove to be another angle by preventing the harmful effects of extracellular ATP on cholangiocytes and the liver. Antagonists to P_2_X_7_, the receptor that triggers inflammasome activation in Kupffer cells, might reduce the inflammatory cytokine surge that damages bile ducts (57, 99). P_2_X_7_ blockers are being studied in non-alcoholic steatohepatitis (NASH) and other inflammatory diseases and could conceivably be used around transplantation to limit macrophage activation (100, 101).

Despite their therapeutic promise, systemic modulation of purinergic signaling is not without potential limitations. A_2_A receptor agonists may induce systemic hypotension, tachycardia, or off-target immunosuppression, while prolonged IL-6 elevation downstream of A_2_B activation carries risks related to maladaptive proliferation and oncogenic signaling (73, 102, 103). additionally, both P_1_ and P_2_ receptors are widely expressed across organ systems, raising concern for unintended off-target effects with systemic administration (104).

These considerations underscore the growing appeal of localized or *ex vivo* delivery strategies. Normothermic or hypothermic machine perfusion platforms offer a unique opportunity to deliver purinergic modulators such as A_2_A agonists, apyrase, or P_2_X_7_ antagonists directly to the graft prior to implantation. Such approaches may maximize biliary protection while minimizing systemic exposure, representing a critical future direction for translating purinergic-based therapies into clinical liver transplantation.

## 5 Conclusion

Purinergic signaling plays a pivotal role in how cholangiocytes and the liver as a whole respond to ischemia and reperfusion. By swiftly converting extracellular ATP to adenosine, the CD39/CD73 axis creates a protective halo of adenosine signaling that can temper the immune attack and support cell survival. Cholangiocytes benefit from this in multiple ways, increasing adenosine receptor stimulation that promote their proliferation and survival. The challenge moving forward is translating these pre-clinical insights into therapies—whether through pharmacological preconditioning, *ex vivo* organ perfusion treatments, or new drugs like soluble CD39. Given the morbidity of biliary complications in liver surgery and transplantation, these purinergic-focused strategies hold significant clinical promise. Thus, targeting these molecules may be a key to protecting cholangiocytes from ischemic insults and improving liver graft outcomes in the future.
